# Acute PM2.5 Exposure in Distinct NSCLC Cell Lines Reveals Strong Oxidative Stress and Therapy Resistance Signatures Through Transcriptomic Analysis

**DOI:** 10.3390/toxics13060484

**Published:** 2025-06-08

**Authors:** Aussara Panya, Saruda Thongyim, Pachara Sattayawat, Sahutchai Inwongwan

**Affiliations:** 1 Department of Biology, Faculty of Science, Chiang Mai University, Chiang Mai 50200, Thailand; aussara.pan@cmu.ac.th (A.P.); pachara.sattayawat@cmu.ac.th (P.S.); 2 Office of Research Administration, Chiang Mai University, Chiang Mai 50200, Thailand; saruda.th@cmu.ac.th

**Keywords:** PM2.5, NSCLC, acute exposure, EGFR mutation, oxidative stress, therapy resistance

## Abstract

Acute PM2.5 exposure has been implicated in lung cancer progression, yet its impact on genetically distinct NSCLC cells remains underexplored. This study investigates how mutation-specific transcriptional responses influence susceptibility to PM2.5-induced oncogenic alterations, focusing on A549 and NCI-H1975 cells. This provides preliminary insight into the transcriptomic effects of acute PM2.5 exposure in NSCLC cells with distinct oncogenic mutations (A549 and NCI-H1975), serving as a guide for understanding mutation-specific responses to environmental stress. Cells were exposed to PM2.5 (200 µg/mL, 24 h), followed by RNA sequencing and analysis. Gene ontology and pathway enrichment analyses were conducted to identify key molecular alterations associated with tumour progression. NCI-H1975 cells exhibited a stronger transcriptional response, with a higher fold change in differentially expressed genes (DEGs), indicating greater PM2.5 susceptibility. Upregulated genes were linked to oxidative stress, carcinogen activation, metabolic reprogramming, and therapy resistance, reinforcing tumour survival under PM2.5 stress. Conversely, the downregulation of tumour suppressor genes suggests immune suppression and potential immunotherapy resistance. This study reveals that acute PM2.5 exposure induces mutation-specific transcriptomic alterations in NSCLC, with EGFR-mutant cells exhibiting heightened oxidative stress, metabolic shifts, and immune evasion. The upregulation of key genes highlights the profound molecular impact of short-term exposure, paving the way for future studies on pollution-driven oncogenic mechanisms and resistance pathways.

## 1. Introduction

Fine particulate matter (PM2.5, particles ≤ 2.5 µm in diameter) is a major air pollutant originating from vehicular emissions, industrial activities, biomass and coal combustion, soil and dust resuspension, and cooking fumes. Epidemiological studies have established a strong correlation between chronic PM2.5 exposure and increased morbidity and mortality from respiratory and cardiovascular diseases, as well as various cancers, particularly lung cancer [1,2,3,4,5,6]. Long-term PM2.5 exposure is known to accelerate lung cancer progression by altering oncogenes and tumour suppressor genes, modifying the tumour microenvironment, and promoting inflammation, angiogenesis, autophagy, and apoptosis, all of which contribute to tumour growth and metastasis [7]. It has also been reported to induce oxidative stress, inflammation, and oncogene activation, including the epidermal growth factor receptor (EGFR) pathway, which plays a key role in tumour progression and is therefore a target for investigation under acute exposure conditions [8,9,10]. Recent studies suggest that PM2.5 enhances NSCLC progression through multiple oncogenic pathways. PM2.5 exposure has been shown to increase cancer cell proliferation, migration, and invasion, inducing epithelial–mesenchymal transition (EMT) and cancer stem cell (CSC) properties [11]. A study on mild PM2.5 exposure revealed increased angiogenic activities in endothelial and NSCLC cells and enhanced lung cancer growth and angiogenesis through hypoxia-inducible factor-1α (HIF-1α) in a xenograft mouse model [12]. Transcriptomic profiling of A549 cells has also identified IL1β and MMP1 as key regulators of PM2.5-induced NSCLC progression, with their expression significantly elevated upon exposure [13]. Additionally, PM2.5 has been reported to activate the aryl hydrocarbon receptor (AhR), which promotes EGFR signalling and upregulates oncogenic factors such as TMPRSS2 and IL18, contributing to tumour progression [9]. These findings indicate that PM2.5-induced physiological changes occur through multiple oncogenic and inflammatory pathways, but the extent of these effects may vary based on genetic mutations. This study compares the transcriptomic responses of A549 (KRAS-mutant, p53 wild-type) and NCI-H1975 (EGFR-mutant, p53-mutant) cells following acute PM2.5 exposure. By identifying differentially expressed genes (DEGs, supplementary materials) and altered pathways, we aim to uncover mutation-driven variations in NSCLC progression under environmental stress, with implications for patients in high-pollution areas.

## 2. Materials and Methods

### 2.1. Cell Culture and PM2.5 Exposure

A549 and NCI-H1975 NSCLC cell lines were cultured in RPMI-1640 medium supplemented with 10% foetal bovine serum (FBS) at 37 °C and 5% CO_2_. Urban PM2.5 was obtained from the National Institute of Standards and Technology (NIST) Standard Reference Material (SRM) 1648a (Urban Particulate Matter; Gaithersburg, MD, USA), resuspended in sterile PBS at 10 mg/mL, and sonicated for 30 min to ensure uniform dispersion. Cells were seeded at 2 × 10^5^ cells/well in 6-well plates, allowed to adhere overnight, and treated with 200 µg/mL PM2.5 for 24 h. Controls received an equivalent volume of PBS. SRM 1648a, a widely used benchmark in in vitro toxicology studies, represents typical urban PM2.5 and contains trace metals, inorganic ions, and carbonaceous matter.

### 2.2. RNA Extraction, Quality Control, and Sequencing

Total RNA was extracted using the Zymo Research Quick-RNA™ MiniPrep Kit (Zymo Research, Irvine, CA, USA) according to the manufacturer’s protocol. RNA concentration and purity were assessed using a NanoDrop 2000 spectrophotometer (Thermo Fisher Scientific, Waltham, MA, USA). RNA sequencing was performed by Novogene Co., Ltd. (Beijing, China), with library preparation and sequencing on an Illumina platform using SBS technology (Illumina, San Diego, CA, USA).

### 2.3. Bioinformatics and Statistical Analysis

Raw reads were filtered to remove adapter sequences, low-quality bases, and reads with >10% ambiguous nucleotides. Clean reads were aligned to the human reference genome (GRCh38) using HISAT2 v2.0.5, a graph-based splice-aware aligner optimized for RNA-seq data. Gene-level expression was quantified using featureCounts v1.5.0-p3, and normalized as fragments per kilobase of transcript per million mapped reads (FPKM). Differential gene expression analysis was conducted using DESeq2 (v1.42.0, Bioconductor) for biological replicates and edgeR (v4.2.0, Bioconductor) for non-replicates. Both tools apply negative binomial generalized linear models and estimate gene-wise dispersions to test for significance. Genes with |log2 fold change| ≥ 1.0 and *p*-value (FDR or *p*) ≤ 0.05 were considered significantly differentially expressed. Functional enrichment of DEGs was performed using the R package clusterProfiler (v4.12.2, Bioconductor) against GO, KEGG, Reactome, Disease Ontology (DO), and DisGeNET databases.

## 3. Results

### 3.1. Global Transcriptomic Profiling of NSCLC Cells Following PM2.5 Exposure

**Figure 1:**
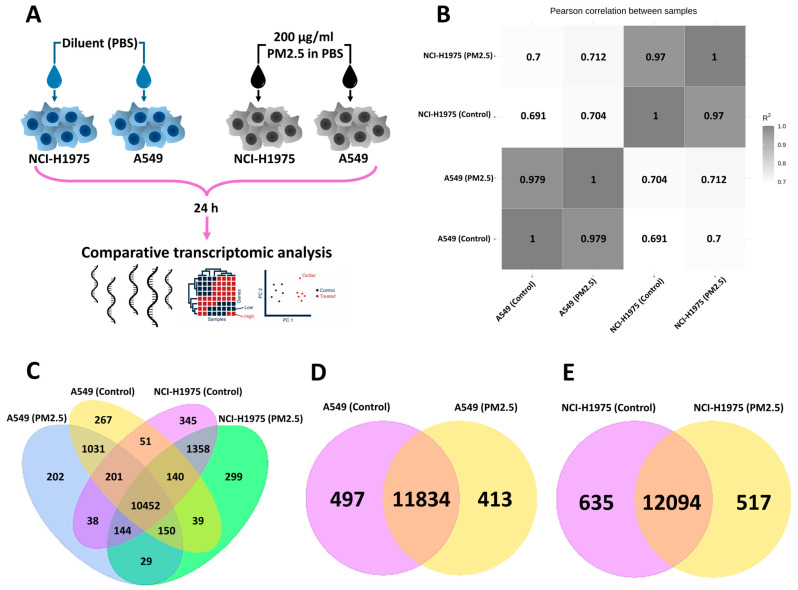
Global transcriptomic changes in NSCLC cell lines following PM2.5 exposure. (**A**) Experimental design showing acute PM2.5 exposure (200 µg/mL, 24 h) in A549 and NCI-H1975 cells. (**B**) Pearson correlation heatmap showing higher similarity within cell types than between treatments. (**C**) Venn diagram displaying shared and unique expressed genes across four groups (A549 control, A549 PM2.5-exposed, NCI-H1975 control, and NCI-H1975 PM2.5-exposed). (**D**,**E**) Gene overlap analysis showing shared and differentially expressed genes in A549 (**D**) and NCI-H1975 (**E**) following PM2.5 exposure.

The transcriptomic analysis of A549 and NCI-H1975 NSCLC cells revealed cell-type-specific responses to PM2.5 exposure (200 µg/mL, 24 h). As shown in Figure 1A, the high-dose exposure model allowed for the identification of key gene expression changes. Pearson correlation analysis (Figure 1B) demonstrated that cell lineage, rather than PM2.5 exposure, was the dominant factor influencing transcriptional profiles, with a strong R^2^ correlation between biological replicates of the same cell type. The four ways Venn diagram (Figure 1C) illustrates gene expression patterns across four groups: A549 (control), A549 (PM2.5-exposed), NCI-H1975 (control), and NCI-H1975 (PM2.5-exposed). A core set of 10,452 genes was shared among all conditions, while 1031 genes in A549 and 1358 genes in NCI-H1975 were uniquely differentially expressed following PM2.5 exposure. Despite a high degree of overlap, exposure led to distinct transcriptional shifts, suggesting environmental stress-induced regulatory changes. When comparing control and PM2.5-exposed cells, most genes remained commonly expressed, with 11,834 shared genes in A549 and 12,094 in NCI-H1975 (Figure 1D and Figure 1E, respectively). However, 400–600 genes were uniquely differentially expressed following exposure, highlighting specific PM2.5-induced transcriptional responses. Housekeeping genes were consistently expressed across all conditions, while DEGs will be further investigated for their roles in cancer progression and environmental adaptation.

### 3.2. Differentially Expressed Genes (DEGs) in A549 and NCI-H1975 Cells Following PM2.5 Exposure

**Figure 2:**
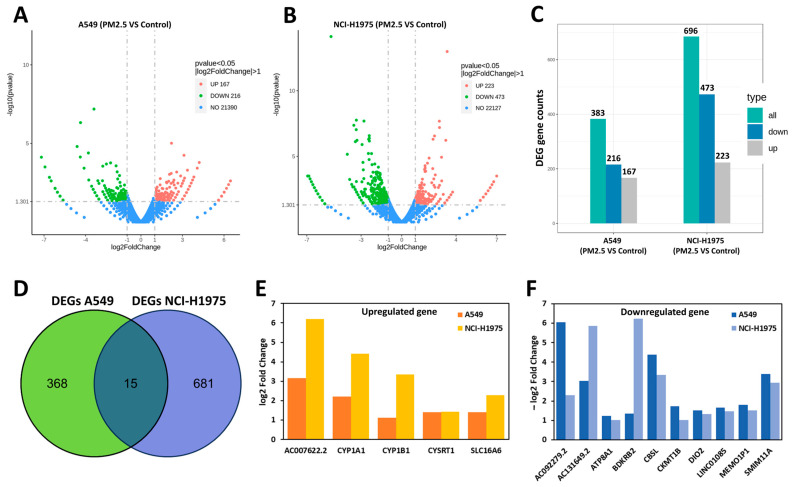
Analysis of differentially expressed genes (DEGs) and expression profiles in PM2.5-exposed A549 and NCI-H1975 compared to control. (**A**) Volcano plot showing DEGs in A549 cells exposed to PM2.5 versus untreated controls. (**B**) Volcano plot showing DEGs in NCI-H1975 cells exposed to PM2.5 versus untreated controls. Significantly upregulated (red) and downregulated (green) genes were identified using *p*-value ≤ 0.05 and |log_2_ fold change| ≥ 1.0 (grey dotted lines); non-significant genes are shown in blue. (**C**) Bar chart summarizing the number of total, upregulated, and downregulated DEGs identified in A549 and NCI-H1975 cells. (**D**) Venn diagram illustrating overlapping DEGs between the two cell lines. (**E**) Representative commonly upregulated genes in both cell lines, with their respective log_2_ fold change values. (**F**) Representative commonly downregulated genes in both cell lines, with their respective log_2_ fold change values.

Transcriptomic analysis revealed distinct gene expression profiles between PM2.5-exposed and control NSCLC cells. Differential expression analysis was performed to compare PM2.5-treated samples directly against their corresponding untreated controls within each cell line. Volcano plots (Figure 2A,B) illustrate the distribution of differentially expressed genes (DEGs), with significantly upregulated and downregulated genes identified based on a *p*-value ≤ 0.05 and |log_2_ fold change| ≥ 1.0. The EGFR-mutant NCI-H1975 cells exhibited a more pronounced transcriptomic response, with 696 DEGs (223 upregulated, 473 downregulated), compared with 383 DEGs in the A549 cells (167 upregulated, 216 downregulated), as summarized in Figure 2C. These findings suggest greater sensitivity to PM2.5 exposure in genetically distinct tumour types. Despite this, the two cell lines shared only five upregulated and ten downregulated genes (Figure 2D), indicating a predominantly cell-type-specific response. Interestingly, while NCI-H1975 had a higher number of DEGs, A549 exhibited a greater magnitude of expression changes, with an average log2FC of ~3.1 per DEG, compared with ~2.6 in NCI-H1975, suggesting fewer but more pronounced transcriptional shifts in A549, whereas NCI-H1975 showed a broader, moderate response. Among the upregulated genes, AC007622.2 had the highest log2FC (3.2 in A549, 6.2 in NCI-H1975). CYP1A1 and CYP1B1, involved in xenobiotic metabolism, increased by 2.2-fold and 1.1-fold in A549 and 4.4-fold and 3.3-fold in NCI-H1975, respectively. SLC16A6 was upregulated by 1.4-fold in A549 and 2.3-fold in NCI-H1975, while CYSRT1 increased 1.4-fold in both cell lines. Among the downregulated genes, CBS decreased by −4.4 in A549 and −3.3 in NCI-H1975, while BDKRB2 showed a stronger reduction in NCI-H1975 (−3.5) than A549 (−1.8). LINC01085 was downregulated by −2.6 in A549 and −3.1 in NCI-H1975, while ATP8A1 (−2.1 in A549, −2.8 in NCI-H1975) and DIO2 (−1.9 in A549, −2.4 in NCI-H1975) also exhibited significant suppression. These findings underscore the complexity of NSCLC responses to environmental stress, highlighting mutation-dependent transcriptional shifts in PM2.5-exposed lung cancer cells.

### 3.3. Pathway Enrichment Analysis of DEGs in A549 and NCI-H1975 Cells

**Figure 3:**
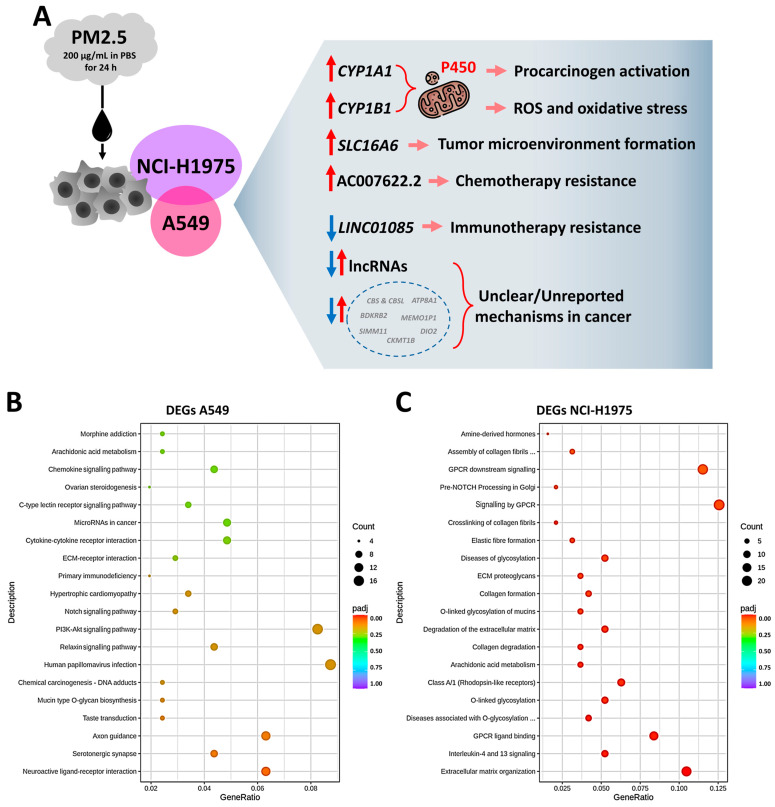
Functional impact of common differentially expressed genes (DEGs) and pathway enrichment analysis. (**A**) Potential effects of commonly DEGs in A549 and NCI-H1975 cells. (**B**,**C**) Pathway enrichment analysis of DEGs in A549 (**B**) and NCI-H1975 (**C**), highlighting key biological processes affected by PM2.5 exposure.

Pathway enrichment analysis revealed distinct molecular responses to PM2.5 exposure in A549 (Figure 3B) and NCI-H1975 (Figure 3C) cells. NCI-H1975 exhibited a stronger enrichment response, with most pathways falling within the lower *p* range compared with A549, indicating higher transcriptional activation in EGFR-mutant cells. In A549 cells, the most significantly enriched pathways included Neuroactive ligand–receptor interaction, PI3K-Akt signalling, and Axon guidance, suggesting roles in cellular communication, survival signalling, and cytoskeletal remodelling. In NCI-H1975 cells, key enriched pathways involved extracellular matrix (ECM) organization, interleukin-4 and interleukin-13 signalling, GPCR ligand binding, *O*-glycosylation disorders, Class A/1 rhodopsin-like receptors, GPCR signalling, GPCR downstream signalling, and ECM degradation. These pathways highlight stronger immune modulation, extracellular remodelling (potentially microenvironmental changes), and signal transduction activity compared with A549. The higher pathway enrichment and lower *p*-values in NCI-H1975 suggest that EGFR-mutant NSCLC cells exhibit a more pronounced transcriptional response to PM2.5, particularly in ECM reorganization, immune signalling, and GPCR-mediated pathways.

## 4. Discussion

This study aimed to elucidate the molecular impact of acute PM2.5 exposure (200 µg/mL, 24 h) on two NSCLC cell lines (A549 and NCI-H1975), each with distinct mutational backgrounds—A549 (KRAS-mutant, p53 wild-type) and NCI-H1975 (EGFR-mutant, p53-mutant). While the PM2.5 concentration used in this study (200 µg/mL for 24 h) exceeds typical ambient levels, it aligns with established in vitro models designed to simulate acute high-exposure events. Such exposures can occur during severe haze episodes—e.g., seasonal biomass burning in Northern Thailand or industrial smog—and in occupational settings such as firefighting or mining, where short-term PM2.5 peaks may reach 500–1000 µg/m^3^ [14,15,16]. High-dose in vitro exposures (100–400 µg/mL) have been widely used to reveal the rapid effects of PM2.5, including ROS generation, DNA damage, and inflammatory signalling [17,18,19]. Our use of an acute model complements chronic exposure studies by capturing early molecular responses to high-dose insults, which may contribute to disease exacerbation and mutation-specific vulnerabilities in NSCLC.

The PM2.5 used in this study was NIST SRM 1648a, a well-characterized urban particulate matter standard widely used in toxicology research. It contains a representative mix of trace metals (e.g., Pb, Zn, and Fe), inorganic ions (e.g., sulphate), carbonaceous material (organic and elemental carbon), and PAHs, closely reflecting typical urban air pollution profiles [20,21,22]. This complexity mirrors ambient PM2.5 found in global cities, supporting its relevance to real-world exposure. While meteorological data were not collected for this standard, prior studies indicate that such components can act synergistically to amplify toxicity, particularly through interactions with co-pollutants such as NOx and O_3_, or lifestyle factors such as smoking [23,24].

In this study, the higher fold change (FC) in EGFR-mutant NCI-H1975 cells suggests that PM2.5 may enhance resistance to EGFR-targeted therapies, aligning with research showing that PM2.5 promotes lung carcinogenesis by inducing inflammation, oxidative stress, and DNA damage, selecting for EGFR mutations that drive oncogene activation, tumour progression, metastasis, and drug resistance, while AhR-Src signalling fosters cancer proliferation and EGFR-TKI resistance [8,9,10]. One of the most highly upregulated genes was AC007622.2, a long noncoding RNA (lncRNA) with a log2FC of 3.2 in A549 and 6.2 in NCI-H1975. This lncRNA has been previously suggested to be associated with cancer resistance to chemotherapy [25]. This finding aligns with numerous reports suggesting that differential expression of lncRNAs significantly contributes to either promoting or inhibiting cancer, playing an extremely complex and ambiguous role in cell physiology [26,27,28]. In this study, we found that CYP1A1 and CYP1B1, prominent members of the cytochrome P450 enzyme family, have been thoroughly investigated for their ability to activate substances with carcinogenic characteristics [29], and have been previously reported to play significant roles in the pathogenesis of lung cancer [30]. Elevated CYP1A1 inducibility is linked to pulmonary PAH-related DNA adduction and an increased risk of lung cancer [31]. CYP1A1, predominantly expressed in extrahepatic tissues, especially the lung, plays a major role as a carcinogen-activating enzyme in the CYP system [32], metabolizing and being induced by polycyclic aromatic hydrocarbons (PAHs), generating highly reactive oxygen species (ROS), which can form DNA adducts and promote tumorigenesis. This corresponds with previous reports that PM2.5 exposure enhances A549 cell invasiveness, increases ROS production, and disrupts mitochondrial function, underscoring its potential role in promoting lung cancer aggressiveness [33]. However, despite their significant upregulation, these genes have not yet been highlighted as predominant drivers of PM2.5-induced transcriptional changes.

PM2.5 exposure not only activated oxidative stress pathways but also significantly altered cancer metabolism and tumour microenvironment formation, as evidenced by the upregulation of SLC16A6, a member of the monocarboxylate transporter (MCT) family involved in the export of lactate, pyruvate, and ketone bodies, all of which play crucial roles in cancer metabolism and microenvironment regulation [34,35]. These genes are key contributors to tumorigenesis and progression, with prognostic significance in lung adenocarcinoma [36]. The SLC16 family underscores its involvement in cancer metabolism, immune infiltration, and tumour stem cell regulation, suggesting its potential as a prognostic marker and therapeutic target. SLC16A6 expression increased by 1.4-fold in A549 and 2.3-fold in NCI-H1975, indicating that metabolic shifts under PM2.5 stress are more pronounced in EGFR-mutant cancer cells. Among the commonly upregulated DEGs, CYSRT1 exhibited a 1.4-fold increase in expression in both A549 and NCI-H1975 following PM2.5 exposure. Although CYSRT1 has not been directly linked to lung cancer, previous studies associate it with oesophageal squamous cell cancer progression [37,38], suggesting a potential role in cancer-associated signalling pathways.

Several key genes were significantly downregulated, indicating a complex cellular response to PM2.5 exposure. LINC01085, a tumour suppressor with potential immunotherapy relevance, was downregulated in both cell types [39]. A study demonstrated that LINC01085 overexpression combined with immune checkpoint blockade is an effective strategy in HIPC patients [40]. While its role in lung cancer remains unclear, its downregulation in PM2.5-exposed cells suggests a potential resistance to LINC01085-based immunotherapies. Additional consistently downregulated genes included lncRNAs AC092279.2 and AC131649.2, CBS, BDKRB2, SIMM11, MEMO1P1, CKMT1B, ATP8A1, and DIO2. While the functions remain unclear, others have been implicated in cancer progression. CBS (Cystathionine-Beta-Synthase), which is involved in tumour metabolism and redox balance, was downregulated, aligning with its subtype-specific roles in lung cancer [41]. BDKRB2, encoding a bradykinin receptor linked to angiogenesis, was also significantly reduced [42,43]. Cir-MEMO1, a pseudogene associated with cell motility, glycolysis, and apoptosis regulation in NSCLC, was suppressed following PM2.5 exposure [44]. CKMT1B, implicated in immune infiltration and glioma prognosis, was also downregulated [45]. Among NSCLC-associated genes, ATP8A1 downregulation suggests a PM2.5-driven reduction in invasion ability [46], while DIO2 downregulation has been linked to shorter disease-free and overall survival in NSCLC patients [47]. These reported genes indicate that acute PM2.5 exposure induces mutation-specific transcriptional changes in NSCLC cells, with EGFR-mutant NCI-H1975 cells showing greater susceptibility due to oxidative stress, carcinogen activation, and immune suppression, underscoring the need for targeted interventions in high-pollution environments. While this study provides transcriptomic evidence of PM2.5-induced alterations in xenobiotic metabolism, oxidative stress, and metabolic transport, the functional validation of key DEGs remains to be performed. Recent work demonstrated that PM2.5 promotes NSCLC progression via DLAT-mediated glycolysis using in vivo and translational approaches [48], emphasizing the importance of such validation. Our findings offer complementary molecular targets for future mechanistic studies.

## 5. Conclusions

This study demonstrates that acute exposure to urban PM2.5 induces significant, mutation-specific transcriptomic alterations in NSCLC cell lines. EGFR-mutant NCI-H1975 cells exhibited a broader and more pronounced transcriptional response than KRAS-mutant A549 cells, with upregulation of genes associated with oxidative stress, xenobiotic metabolism, metabolic reprogramming, and immune modulation. Notably, the marked activation of CYP1A1, CYP1B1, and SLC16A6, coupled with the downregulation of tumour suppressors such as LINC01085 and CBS, underscores the potential of PM2.5 to promote oxidative stress, carcinogenesis, and therapy resistance in a mutation-dependent manner. These findings suggest that individuals harbouring specific oncogenic mutations may be more susceptible to air-pollution-induced tumour progression and immune evasion. Although further functional validation is warranted, our results offer important insight into the molecular mechanisms of pollution-associated lung cancer aggressiveness and highlight potential targets for therapeutic intervention and biomarker development, particularly in high-exposure settings.

## Data Availability

Data are contained within the article and upon request. The complete coding sequence (CDS) RNA and DEGs data can be found in the Supplementary File.

## Supplementary Materials

The following supporting information can be downloaded at: https://www.mdpi.com/article/10.3390/toxics13060484/s1.
